# The Future of Evidence-Based Temperance Interventions

**DOI:** 10.3389/fpsyg.2021.707598

**Published:** 2021-08-13

**Authors:** Everett L. Worthington, Llewellyn E. van Zyl

**Affiliations:** ^1^Department of Psychology, Virginia Commonwealth University, Richmond, VA, United States; ^2^Department of Industrial Engineering and Innovation Sciences, University of Eindhoven, Eindhoven, Netherlands; ^3^Optentia Research Focus Area, North-West University (VTC), Vanderbijlpark, South Africa; ^4^Department of Human Resource Management, University of Twente, Enschede, Netherlands; ^5^Department of Social Psychology, Institut für Psychologie, Goethe University, Frankfurt am Main, Germany

**Keywords:** positive psychological interventions, temperance, forgiveness, future perspectives, humility, patience, positive psychology

## Abstract

Positive psychology has accumulated a large and ever-growing body of scientific knowledge about human strengths and virtues. However, research on positive psychology interventions (PPIs) to develop such is still in its infancy. In this brief position paper, we summarize the status of PPIs in one of the positive psychology’s most important virtues: temperance. Temperance refers to the capacity to manage habits and protect against excess and is composed of forgiveness, humility, and (we include) patience. Specifically, we examine the current state-of-the-science in the conceptualization of temperance, explore the efficacy of temperance interventions, and reflect upon what the future may hold in this research domain. In this paper, we first highlight the challenges and opportunities for expanding the theoretical conceptualization of temperance and reflect upon the challenges in temperance-related PPIs. For each aspect of temperance, we propose a specific research agenda. Second, we explore what is needed for PPIs to promote temperance and how growth in temperance intervention research can be fostered. Generally, while forgiveness interventions are well established, we recommended that both humility and patience interventions need more viable evidence-based research on existing and new interventions. Third, we advanced several recommendations regarding how to promote more research in new interventions. These recommendations included attracting more funders to the area, developing new interventions, and employing new technology. Because intervention research in temperance is in its infancy, the future looks rosy for PPI researchers as we move into a second generation of positive psychology research.

## Introduction

Positive psychology has accumulated a large and ever-growing body of scientific knowledge about character strengths and virtues (Van Zyl et al., 2021). However, research on positive psychology interventions (PPIs) to help people develop their character is still in its infancy (Van Zyl et al., 2019). In this brief position paper, we evaluate the status of PPIs in one of the positive psychology’s most important virtues—temperance. Specifically, we examine our perspective on the current state-of-the-science in the conceptualization of temperance, explore the efficacy of temperance interventions, and reflect upon what the future may hold in this research domain. Importantly, this is not a systematic review of the literature. Rather, we have drawn on our baseline knowledge of the literature and offered our perspective. We hope that our perspective might be heuristic and stimulate meta-analyses, systematic reviews, and empirical research.

Each virtue is important, and in many ways, all virtues are interconnected. However, some seem more fundamental than do others because they organize or empower others. For example, humility, which is not strictly a virtue in Peterson and Seligman’s (2004) category system, traditionally has been seen as a virtue and some philosophers (i.e., Augustine) have named it as the most central virtue because it is an attitude that orients one toward goodness or virtue. Self-control or self-regulation also is seen as an essential virtue because it is methodologically central. That is, most virtues involve inhibiting natural proclivities toward self-interest or even vice. Self-regulation or self-control is methodologically how this is often done. Finally, wisdom is a virtue needed to discern among competing goods. There is also an empirical basis for seeing temperance (involving self-regulation and the ability to focus wisely on others) as central (see McGrath and Walker, 2016).

## Temperance—Its Components and Possibilities

Peterson and Seligman’s (2004) classified temperance as one of the six universal virtues they identified as being cross-culturally valued, leading to a genuinely good character. It is also a fundamental component of leading a happy, healthy, and flourishing life (Worthington, 2020a). This virtue reflects an inherent capacity to moderate or control one’s thoughts, feelings, habits, and desires (Mayerson, 2020) that protects against excess or deficiency (Fehr and Gelfand, 2012). Temperance may encompass many related behaviors, such as prudence, conscientiousness, caution, and self-restraint, that could tame impulses of anger, resentment, selfishness, over-indulgence, and rigidity (Shahab and Adil, 2020). According to Peterson and Seligman (2004), as a virtue of good character, temperance can be exerted through four signature strengths: *forgiveness and mercy*, *humility*, *prudence/self-regulation*, and (we would add) *patience* (Schnitker and Emmons, 2007). Peterson and Seligman (2004) included prudence/self-control as their last two character strengths making up the virtue of temperance. Prudence is classically considered the ability to govern oneself by reason (which seems like one form of self-control—cognitive), and self-control is also considered self-governance—and is related to self-regulation (of cognition and behavior), emotion regulation, and other ways of stifling unwanted experiences and driving desired but hard-to-do experiences. Thus, prudence and self-control seemed confounded and not distinct in Peterson and Seligman’s taxonomy. Furthermore, little empirical evidence exists in actively developing prudence and self-regulation through PPIs (Van Zyl and Rothmann, 2019). There is thus a pertinent need to theoretically and empirically explore these components from a positive psychological perspective and invest more resources in clarifying its role within the positive psychological lexicon (Summers, 2020). As such, we do not consider these factors further in our paper. With the exclusion of prudence/self-regulation, all of these factors have enjoyed extensive empirical attention in the positive psychology literature and formed the basis of several important PPIs (Worthington, 2019). We, therefore, choose to focus on the qualities of forgiveness, humility, and patience and the PPIs that promote them.

### Forgiveness and Mercy

Forgiveness refers to a form of intraindividual, pro-social change that occurs within an individual that changes one’s attitude toward a transgressor to become more benevolent and less vengeful/avoidant (Worthington, 2020a). Conceptually, a distinction should be made between “forgivingness” and “forgiveness.” The former pertains to an individual’s dispositional readiness to show forgiveness, whereas the latter refers to the psychological changes that take place which changes the perception of the transgressor or transgression (Peterson and Seligman, 2004). Forgiveness is also conceptually different from reconciliation. Where forgiveness is focused on changing one’s feelings and behavioral intentions toward a transgressor, reconciliation refers to the extent toward which a damaged interpersonal relationship can be repaired through mutual, trustworthy, and supportive behaviors (Worthington and Drinkard, 2000).

Forgiveness should also not be confused with mercy. Whereas forgiveness is an “internal process,” mercy is an act by one with authority to administer or pursue less punishment than is justly deserved (Worthington, 2006). Forgiveness is, therefore, a “*general concept reflecting kindness, compassion or leniency toward (1) a transgressor (2) someone over whom one has power or authority or (3) someone in great distress*” (Peterson and Seligman, 2004, p. 446). Fundamentally, forgiveness involves changes in emotions and behavioral intentions, which result in perceptions of the transgressor/transgression to become more positive and less negative (Worthington, 2006). In other words, the transgressor is viewed with more understanding, benevolence, compassion, and kindness (Shahab and Adil, 2020) and less hatred and vengeance is felt (Worthington, 2020a).

Forgiveness is therefore composed of two experiences that are related but not intertwined inextricably. *Decisional forgiveness* is a behavioral intention to treat a transgressor as a valued and valuable person and not pursue revenge for an offense (Worthington and Wade, 2020). Decisions to forgive can be followed through on and yet emotionally, the decisional forgiver could still feel resentment, bitterness, hatred, anger, and anxiety (i.e., unforgiveness). Thus, a second type of forgiveness, *emotional forgiveness*, must exist. Emotional forgiveness is the replacement of unforgiving emotions and motivations with other oriented, positive emotions (i.e., empathy, sympathy, compassion, or love) and benevolent motivations (Worthington, 2006). Berry, Worthington et al. (2001) argued that forgiveness is largely an affectively experienced phenomenon, where experiences of resentment, bitterness, and the like are neutralized through empathy, kindness, compassion, and humility.

Although forgiveness research has enjoyed much empirical attention over the last century, we believe there are still a number of conceptual issues needing clarification. In this brief perspective article, we admit to the inadequacy of being able to justify each recommendation in the present bulleted list (and the bulleted lists for humility and patience) within an essay of reasonable length. We generally made our recommendations because (1) they had not been previously researched and (2) they seemed to us to be needed, given our subjective sense of what might be needed to push ahead research on each topic. We hope our suggestions are heuristic, and we apologize for the lack of detailed context around each one. With that caveat in mind, we believe the next decade in forgiveness research should as:

- Determine the growth-promoting and transcendence functions of forgiveness, neither of which have received much research given the focus on the deleterious effects of not forgiving characterizing much research (Worthington, 2020b);
- Find differences in the ways that decisional and emotional forgiveness interact with each other over time and ways that each contribute to spiritual, relational, psychological, and physical health outcomes; whereas the two types of forgiveness have been hypothesized to have different spiritual, relational, psychological, and physical health outcomes, little systematic research has investigated what those differences are (Worthington, 2020b);
- Enhance and supplement theoretical and empirical frameworks for self-forgiveness; Woodyatt and Wenzel (2020) have systematically reviewed the research and arrived at a similar conclusion;
- Map interconnections, especially over time, among decisions to forgive, emotional forgiveness, observing in offenders expressions of accountability (e.g., remorse, regret, apology, and amends making), and observing in offenders indications of self-forgiveness and feeling forgiven by God (Worthington, 2020b);
- Explore context-specific variables that affect the presence or absence of forgiveness in dyadic, family, group, community, and macro (i.e., cultural) contexts (for reviews, see Worthington and Wade, 2020); and
- Investigate the future cultural value of forgiving (qua justice) in this era of increasing political and ideological polarization that has resulted in a strong valuing of punitive justice of those who harm and censure of those failing to support social justice for those who lack social power (Sandage et al., 2020).

### Humility

Humility is a character strength rooted in the work virtuous ethics. It is a critical component of relationships providing social oil that facilitates smooth relationships and a buffer against hostility and relationship-disintegration when things go wrong in relationships. Humility also directly affects mental health and wellbeing (Worthington and Allison, 2018), and it often acts through relationships to further affect mental health and wellbeing. From this perspective, humility has been defined as a non-defensive inclination to view the self accurately or realistically, including both one’s strengths and developmental areas (Peterson and Seligman, 2004). Recent conceptualizations of humility are considerably more refined than initial research conceptualizations. Humility occurs when individuals let their accomplishments speak for themselves, and actively refrain from distorting information to defend, verify or repair their own personal image (Watkins et al., 2016). As a foundational virtue, Worthington and Allison (2018) argued that humility is composed of three necessary and sufficient conditions. Humility must include (1) an accurate awareness and modest portrayal of one’s strengths and weaknesses (realistic self-assessment) (2) an authentic appreciation for the strengths and contributions of others (other orientation), and (3) an openness for learning, constructive feedback, and new ideas of others (teachability), all of which quell self-focus. Therefore, expressed humility is discernible (imperfectly) from observable behaviors that manifest during interpersonal interactions.

Humble individuals engage in ongoing *realistic self-assessment* through interactions with others. Humble individuals process information gathered in social interactions as a means to both explore their personal identities (e.g., personality, strengths, and limitations) and to modify their behavior when necessary. Humility, therefore, fosters an accurate or objective appraisal of one’s own strengths and areas needing additional development through a transparent (situation-appropriate) disclosure of their own limitations, openly acknowledging their mistakes, and actively seeking constructive feedback on their own development. Humble individuals tend to show high levels of relational functioning, psychological wellbeing, and mental health (Owens et al., 2013; Watkins et al., 2016; Worthington and Allison, 2018).

When humble individuals show an openness for learning, express an active desire to solicit constructive feedback on their own development, and seek new ideas of others, they show their “*teachability*” (Owens et al., 2013). Open-mindedness, a willingness to learn, and a receptive mindset are, therefore, vital elements of humility. Individuals showing high levels of teachability also afford others an opportunity to share their voices and foster greater trust, motivation, and a heightened sense of justice, fairness, and equity in teams (Cropanzano et al., 2007).

Humility is also a function of an *appreciation for the strengths, value, and contributions of others* (Owens et al., 2013), not merely a decrease in positive appraisal of the self (Worthington and Allison, 2018). Humble individuals can transcend the natural comparative-competitive response when engaging with others. Instead, they aim to acknowledge and celebrate the strengths/contributions of others (Worthington and Allison, 2018). In essence, this others orientation is a physical manifestation of the individual’s positive view of human nature and others (Owens et al., 2013).

Worthington and Allison (2018) suggested that several distinct forms of humility exist. *General trait humility* is a personality disposition (relative to states of humility, such as entering a negotiation with humility rather than arrogance). *Relational humility* is humbleness that is differentially experienced in different relationships with others such that, for example, being a humble boss or worker would entail different manifestations. *Cultural humility* is humbleness about cultural beliefs, differences, and views. *Intellectual humility* is humility about personal opinions, ideas, and intellectual capabilities that one is emotionally invested in. Finally, *spiritual humility* is humility before what one considers sacred.

Although humility has over 300 studies investigating it, it is still concept within positive psychology that requires further exploration. We therefore see a number of possibilities that could strengthen then scientific foundation of humility research. Future research on humility should focus on:

- Clarifying the definition; about half of the researchers see the definitional point of being other oriented to be debatable; and many favor “a quiet ego” as a necessity. Future research should resolve whether that point is affected by collectivistic versus individualist cultures or relationships, by religious beliefs (i.e., Buddhism and Hinduism might favor quiet ego; the Abrahamic faiths might favor other orientation). Such distinctions have not been addressed. While there might be religious and cultural origins to seeing humility as requiring a “quiet ego” or other orientation, it is unclear whether there is any residual religious basis for differing on this crucial point of the definition of humility;
- Articulating an evolutionary theory of humility’s benefits to the species. There are no evolutionary accounts of humility in the literature;
- Describing how humility (with its emphasis on lifting others up), when applied to disadvantaged minorities, is not a call to accept oppression, but rather is a call to social justice. This has long been a criticism of humility from theoreticians writing about implications for people of disempowered status (for essays and debates on this point, see Collins et al., 2020);
- Documenting mental and physical health benefits of humility, whereas the social benefits of humility have been well documented, the mental health and physical health benefits have not been as often studied (see Worthington and Allison, 2018);
- Providing a strong theoretically grounded meta-analytic review of the field. Some systematic reviews exist (i.e., for reviews, see Worthington et al., 2017), but no recent meta-analysis exists; and
- Showing the relationship of humility to other virtues it is and is not related to.

### Patience

Peterson and Seligman (2004) conceptualized patience not as a distinct virtue, but as an amalgam of the virtues of persistence (which they saw as part of courage), open-mindedness (which they saw as part of wisdom and knowledge), and self-regulation (which they saw as a synonym for self-control). Schnitker and Emmons (2007) sought to modify the Peterson and Seligman conceptualization of patience. They showed that all 24 strengths included in the Values in Action Inventory of Strengths (Peterson and Seligman, 2004) accounted for only 26% of variance in patience scores. Thus, Schnitker and Emmons argued that patience was a distinct and separate virtue. Schnitker (2012, p. 263) defined patience as “the propensity of a person to wait calmly in the face of frustration, adversity, or suffering”. We might speculate that the reason that Schnitker and Emmons (2007) found patience to be substantially different from the other virtues Peterson and Seligman (2004) identified resides in *calm* waiting. Self-control or self-regulation could be effective whether the person grumbled, complained, frantically exerted effort, or reluctantly tolerated discomfort or frustration. Certainly, patience can involve persistent calm waiting, but again, most persistence is not characterized by patience; often sheer doggedness is needed for persistence. Finally, open-mindedness can be part of patience, but often it is not; much of patience involves putting up with frustrations, unpleasant events, or small hassles, none of which require much open-mindedness. But, calmness is the aspect of patience that distinguishes it from both self-control and prudence. We have sought to examine patience as part of temperance, complementing forgiveness and humility, even though patience does not fit neatly into Peterson and Seligman’s conceptualization of virtues or character strengths (Schnitker et al., 2017b).

At its core, patience is a hybrid personality disposition that requires a self-transcendent narrative where suffering has meaning, serves a purpose, or can be justified (Schnitker et al., 2017b). McAdams and Pals (2006, p. 206) stated that “patience emerges when characteristic adaptations related to regulating emotions are imbued with a particular narrative that becomes a part of one’s identity.” Building on this definition, Schnitker (2012) developed a three-factor model for patience. Patience is composed of (1) *interpersonal patience* (i.e., a calm response to others perceived as being unpleasant, frustrating, or burdensome), (2) *life hardship patience* (i.e., a calm response to and tolerance for unpleasant or frustrating life events, such as financial hardships), and (3) *daily-hassles* patience (i.e., a calm response to hassles in daily life, such as traffic jams). Managing suffering at various levels from intense pain to aggravating unpleasantness is therefore a core component of patience. The magnitude and severity of the causes of the suffering to be endured are objectively different among these types of patience. However, all three types of patience involve an individual’s calm response to what is subjectively perceived as a source of minor or major suffering (Schnitker et al., 2017b).

All three types of patience have been associated with various positive life- and work-related outcomes (Cagande et al., 2020). High levels of patience have been associated with higher agreeableness, mindfulness, goal effort, progress on goals, and satisfaction with achieving goals and lower neuroticism and anxious attachment (Schnitker, 2012; Cagande et al., 2020; Ng et al., 2021). In addition to the commonalities, *interpersonal patience* was correlated with higher self-esteem, hope, and life satisfaction and lower avoidant attachment and loneliness (Schnitker et al., 2017b). *Life-hardships patience* was found to be correlated with higher conscientiousness, hope, and self-esteem (Schnitker et al., 2017b). *Daily-hassles patience* was also found to be correlated with higher openness and life satisfaction and lower avoidant attachment and depression (Schnitker, 2012).

Despite these positive associations, much work is needed to further differentiate patience from other psychological constructs. Several challenges must be resolved to advance patience research:

- Within the current positive psychological nomological lexicon, it is not yet clear whether patience is to be treated as a strength or a virtue.
- Trait and state patience might well have different sequelae. These have not previously been investigated.
- A clearer distinction between patience and other aspects of personality (such as self-control, conscientiousness, and emotional regulation) needs be established for it to thrive as a stand-alone strength (Schnitker, 2012).
- Because calm waiting, rather than mere waiting, is hypothesized to be essential to patience, experimental determination of how calm one must be and how that is manifested physiologically, cognitively, and behaviorally need to be mapped (for a discussion, see Schnitker et al., 2017b).
- For patience interventions to gain traction, an understanding as to how patience contributes to and complements the virtue of temperance has not been articulated and thus is also needed.

## Efficacy of Temperance Interventions

Numerous interventions have been developed to promote these three aspects of temperance (c.f. Worthington and Wade, 2020). The need for these interventions stems from people’s inherent struggle to forgive, act humbly when they wanted to do so, and wait patiently for good things to develop (Worthington et al., 2014). Thus, many people have sought to build forgiveness, humility, and patience, and providing evidence-based interventions for each aspect of temperance has been an important public service of psychology (Worthington and Wade, 2020). Generally, across the three, the interventions have been found to be efficacious and occasionally effective in common use. We must be cautious in concluding that the interventions have been successful because the intervention research, in general, has several reasons to proceed carefully. First, many more studies have investigated students whose problems have not reached clinical severity. Whereas that suggests wide applicability of psychoeducation, it does not give as much support for clinically severe symptoms. Second, across the three aspects of temperance, there is wide variability in the confidence available for assessing the constructs. Assessment of forgiveness has been found to be excellent (Worthington et al., 2014) and similarly for general humility (see McElroy-Heltzel et al., 2019). However, much fewer assessments (with correspondingly less evidence supporting estimated reliability and validity) have been developed for patience (Schnitker et al., 2017a). Third, whereas numerous interventions to promote forgiveness have been subjected to randomized controlled trials (RCTs; Wade et al., 2014), few interventions have been developed for both humility and patience (Schnitker et al., 2017a; Worthington and Allison, 2018). Thus, it is unclear whether good exercises have yet been developed to help people develop those two aspects of temperance.

In the following brief review, with the intent of uncovering new research directions, researchers generally used similar definitions across each aspect. In addition, the measures used to assess change have been generally the same within each domain.

### Forgiveness Interventions

In many ways, forgiveness might be considered an exemplar for PPIs. Wade et al. (2014) identified 62 RCTs of forgiveness interventions spanning five decades. Wade and Tittler (2020) further found since their original meta-analyses, an additional 16 RCTs were published between the meta-analysis and their review (i.e., 2012–2018). Wade and Tittler (2020) argued that additional field studies and other non-experimental forgiveness interventions—both of which were more applicable to effectiveness than efficacy—were not included in the meta-analysis and review. These need to be documented as well as the RCTs for a more complete picture of the support for forgiveness interventions. Arguably, one reason that forgiveness interventions have been effective and popular has been that they did not focus entirely on its positive psychology potential. When Seligman and Csikszentmihalyi (2000) created positive psychology, they conceptualized it as the positive half of psychology. That is, helping heal dysfunction and problems—something positive in itself, though it focused on getting rid of negative aspects of life—had been the almost sole focus of psychology since World War II, and the founders of positive psychology challenged psychologists to investigate the other half of psychology (Wade et al., 2014). Forgiveness was an ideal bridge between positive psychology and traditional psychology. People who struggled with unforgiveness were clearly troubled, and unforgiveness was wrapped up in serious psychological dysfunction. So it dealt with healing (though unforgiveness was not diagnosable in itself; Worthington, 2020a). But forgiveness was also seen as a virtue, and it therefore also dealt with the positive half of psychology—promoting flourishing (Worthington, 2020b). Some of the major motivations for studying forgiveness psychologically were that it could prevent and heal stress-related physical and mental health disorders, heal relationship ruptures like affairs, incest, and abuse, and help deal with spiritual disruptions (Tittler and Wade, 2019).

Despite differing approaches among commonly studied programs to promote forgiveness, virtually all forgiveness interventions share common elements: (1) they act as conduits for victims to reflect upon and disclose the details of a transgression (2) they aid in altering or reframing the perspective through which transgressions are interpreted (3) they seek to develop empathy, kindness, and understanding for the transgressor, and (4) they promote intentional commitment to forgive transgressors (although this may take time; Wade and Worthington, 2005). Wade and Tittler (2020) found that most forgiveness interventions have aimed at forgiving hurtful offenses, and most effective interventions have taken several hours.

Two theoretical models for promoting forgiveness have dominated as: Enright’s forgiveness process model (Freedman and Enright, 2020) and the REACH Forgiveness model (Worthington, 2020b). But other well-studied interventions include Forgive for Good (Luskin, 2001), emotionally focused therapy (Greenberg and Meneses, 2020), and forgiveness after affairs (Baucom et al., 2009). No single approach has been shown to be more efficacious per hour of treatment. A reliable finding from Wade et al., 2014 meta-analysis is that a linear relationship exists between the effect size of forgiveness (*d*, the “response”) in standard deviations and the time spent conscientiously trying to forgive (*T*, i.e., the “dose”). This dose–response relationship has been described using a regression equation, *d* = 0.124 + 0.046^*^ *T*. The initial intercept is likely a positive effect from hearing a definition of forgiveness and a summary of the relational, psychological, and physical health benefits of forgiveness, which have been rigorously accumulated from basic research on forgiveness. Forgiveness interventions have been beneficial for increasing experiences of forgiveness, hope, optimism about the future, happiness, and wellbeing (Wade and Tittler, 2020). They also have profound long-term effects by decreasing depression and anxiety (Wade et al., 2014). In the future, several goals are important for forgiveness interventionists. First, forgiveness interventionists need to create briefer interventions that cause effects that are substantially above the regression line—i.e., more effective per hour than existing interventions. Second, attention is needed to effectiveness research. Third, interventions need to be scaled up that will entail other challenges. For example, scaling generally produces less impact on the average than does effectiveness research, which is less impactful than efficacy research. Although efficacy studies have progressively gotten larger, none have approached a larger dissemination trial.

### Humility Interventions

For humility interventions, the PROVE Humility model (Lavelock et al., 2014, 2017) and Cuthbert et al.’s (2018) approach with pastors have been evaluated and are evidence-based. The PROVE Humility model was found to be efficacious in two independent trials with undergraduate students. A 7-h PROVE Humility workbook was evaluated against a non-action control in each of the studies by Lavelock and her colleagues. Both studies found changes in pre-post measures of trait humility, trait forgivingness, trait patience, and dispositional emotional negativity. When participants were recruited for a study to build virtues and randomly cast into one of the six conditions (*N* = 208; 32 to 37 per condition)—humility, forgiveness, patience, self-control, positivity, and no-action—the undergraduates who completed a DIY workbook on PROVE Humility (*n* = 26) outperformed a non-action control condition (*n* = 33). When participants were recruited specifically into a study to promote humility, the gains for the workbook (*n* = 39) relative to the control condition (*n* = 33) were about twice as big. Although the results of the interventions were promising, the studies were both with students at a single institution. Cuthbert et al. (2018) motivation, and a heightened sense of justice, fairness, and (2018) did not find efficacy for the humility condition (*n* = 41) beyond a wait-list control condition (*n* = 30) in their study of religious leaders. Cuthbert et al. (2018) workbook involved 16 exercises completed with a partner. One likely reason that no gains were found for the religious leaders is that those leaders were already high in humility, and there was a ceiling effect. At this point, humility interventions have mixed evidence supporting their efficacy, but the variety of interventions is too restricted to evaluate their potential.

Humility interventions appear more difficult for which to recruit participants than are forgiveness interventions. When people struggle with forgiveness, they feel a need. Grudges are unpleasant and revenge motives might frighten people, so they seek ways to eliminate those experiences and forgiving is one salient possibility. However, most people do not consider a deficit of humility to be a problem. In fact, one might be arrogant, and thus conclude that one does not need humility, already perfectly having that strength. One might exhibit too many self-effacing behaviors and feel oneself to be a doormat, but low self-worth might make the person feel unworthy of pursuing humility. Thus, the “market” for humility interventions might be within communities that value humility. These might be religious organizations. But some business organizations operate using a strong ethic of teamwork, based on other-oriented humility. Paradoxically, even armed forces, which train soldiers to be loyal to the small group, might benefit by humility interventions. Finally, Collins (2001) has found that Level 5 business leaders are, at root, humble. So, leadership training might come to embody humility interventions. While there might be several needs for humility interventions, it is likely that the field will build slowly, testing the patience of humility researchers.

### Patience Interventions

Patience interventions have been studied more frequently than have humility interventions. Many interventions with adolescents to promote patience are brief and are aimed at stimulating better performance on frustrating tasks, like writing with the non-dominant hand (Schnitker and Emmons, 2007). Interventions seeking to promote patience have more often been aimed at children or adolescents than at adults. One problem inhibiting both basic and intervention research on patience (Hauser, 2019) is that sensation-seeking peaks earlier in adolescence (17–18) than does self-control (23–26). Their curves, on the average, tend to cross-over about age 22. Volitional processes that strengthen self-control are attention, monitoring, planning, persevering, and inhibition. These rely on executive functioning. Impulsive processes, which can derail self-control, include heightened sensitivity to rewards, risk-taking, a present-oriented (vs future-oriented) perspective, and specific addictions and cravings. Hauser (2019) reviewed research on self-control, including patience. They recommended two general ways of strengthening patient self-control—(1) understand how it works and (2) develop interventions in three categories of treatment. Those categories include practicing, goal attainment, and mental transformation of the situation.

Schnitker (2012) delivered a more complete, yet still brief, psychoeducational patience intervention over 2 weeks in four half-hour sessions to groups of three to six undergraduates, almost all of whom were under 26 years old. The content drew from interventions on meditation, treating hostility in Type A behavior, cognitive behavioral therapy, and promotion of character strengths. Undergraduates (*N* = 71; 61 female) experienced an end-of-treatment increase in trait patience and positive affect but not a decrease in negative affect and a decrease in depression. Gains in trait patience, however, were not maintained at follow-up.

Lavelock et al. (2017) provided the most comprehensive treatment to promote patience yet tested. They randomly assigned undergraduates to 7-h DIY workbook treatments, one of which was to promote patience in the SPACE for patience workbook (i.e., S = Serenity, P = Patient listening and Perspective, A = Allow boredom, C = Comfort with delays, and E = Endure with perseverance). The SPACE for patience DIY workbook (*n* = 28) produced more trait patience, trait self-control, and forgivingness than initially, relative to the control condition (*n* = 33).

Yet many adults over 26 years old struggle with failures in patience. So, assuming maturation will infallibly occur by age 26 is not a fruitful strategy for many. We also must help mature adults to build up a habit of experiencing patience. The interventions created by Schnitker et al. and Lavelock et al. (2017) and tested on college students could provide two PPIs that might work with adults older than 26. Two other interventions that were not explicitly designed to promote patience did produce some increase in patience in adult (over 26) samples. PREP’s 20-h Within My Reach and Within Our Reach marriage and relationship education curricula (Daire et al., 2012) promoted marriage communication skills and showed modest increases in patience. A 40-h Crisis-Intervention Training program (Hanafi et al., 2008) for law enforcement officers promoted some increases in patience as well.

## The Future of Temperance Interventions

Despite significant advancements in establishing temperance as an important concept within the larger nomological network of psychology, and the magnitude of PPIs aimed to develop its core components, we believe that two core questions remain unanswered:

- What is needed for interventions to promote forgiveness, humility, and patience?
- How do we promote growth in temperance intervention research?

We hope that evidence-based temperance PPIs can move beyond efficacy research to effectiveness research and from effectiveness research to scaled up dissemination methods by answering these two questions. We briefly reflect upon each of these questions in a hope to stimulate future research.

### What Is Needed for Interventions to Promote Forgiveness, Humility, and Patience?

#### Forgiveness Interventions

Forgiveness interventions are well proven at the small, efficacy-study level (Wade et al., 2014). They require scaling in size and scope, moving outward to different cultural venues, expansion in terms of types of delivery systems—no longer exclusively focusing on the psychoeducational group, the individual psychotherapy or counseling intervention, or DIY workbooks. Rather, these interventions must be translated into common languages and culturally adapted to become more available for a wider audience.

Another factor to consider is the contextualization of forgiveness interventions. Wade and Tittler (2020) argued that the vast majority of forgiveness interventions had been conducted within Western, educated, industrialized, rich, and democratic (WEIRD) contexts and limited is known about how these could affect people from marginalized communities (e.g., LGBTQ community) or those from non-WEIRD contexts. An over-reliance on the WEIRD not only limits the generalizability of the results but also influences the intervention’s applicability (and adoption) within other contexts. For example, the decolonization of science movement in South Africa is largely centered around the idea that western values and approaches are not usually applicable in African contexts, especially when they negate local values and traditions (De Jong et al., 2018; Heleta, 2018). Not developing interventions within these contexts and local traditions would therefore (out of principle) lead to such being received with skepticism or even outright rejection by these societies. Additionally, Lamb (2005) argued that not considering cultural and sexual identity could also lead to psychological harm being inflicted by forgiveness PPIs.

Forgiveness interventions should, therefore, not only focus on developing forgiveness within the full interpersonal context but also as applying in intergroup contexts as one means to enhance its global applicability (Worthington and Wade, 2020). It is imperative to understand whether suffering caused by a transgressor is seen as a function of an intergroup process or system embeddedness, as opposed to (as has dominated research and treatment through 2020) a response to interpersonal pain (Tittler and Wade, 2019). Therefore, forgiveness interventions should incorporate learnings from both cross-cultural psychology and social-identity theory (Worthington and Wade, 2020). Those modifications might facilitate intergroup forgiveness. Here, future research should focus on the identification of specific cultural- and identity-related moderators that can facilitate or hamper the development of forgiveness. In addition, forgiveness happens in context, so processes involving other actors than the forgiver—like offenders, witnesses, affected family or friend members, communities, and even larger societal actors like activists and politicians—must be incorporated in a full understanding of how to intervene to help the most people (Tittler and Wade, 2019).

Furthermore, even with evidence-based psychoeducation, psychotherapy, couple therapy, and prevention interventions, effectiveness studies are also needed. Rather than continuing to produce additional closely controlled, lab-based efficacy studies in cultures that have already been investigated, controlled effectiveness studies in real-world contexts are needed.

Dissemination studies are also needed in which organizations adopt a particular approach to forgiveness and investigations must be conducted to monitor systems (not merely individual practitioners) to ensure fidelity of delivery and investigate the effects on the entire system (McHugh and Barlow, 2012). Scaling up smaller studies are always fraught, and a common finding is that scaled interventions are not as impactful as are RCTs—whether efficacy or effectiveness trials. Scaled studies engage people who really have little interest in the intervention, and yet get swept into the application of the intervention. Thus, such unmotivated people dilute outcomes. Studies that scale forgiveness interventions into dissemination trials must give particular attention to how to engage people who are initially unmotivated.

Furthermore, shifts in the demand for forgiveness interventions would affect both consumers and creators. Both will be looking to create or engage in new, brief, punchy, emotionally engaging, and motivating interventions that frequently engage the consumer in providing feedback and receiving level-up feedback to reward continued participation (Kelders et al., 2020). This will put a strain on forgiveness-related PPIs. Forgiveness interventions have been shown to be strongly related to amount of time spent trying to forgive (Worthington, 2020a). Effective forgiveness might not be amenable to four-minute, self-administered mini-interventions. Getting participation in completing a 7-h forgiveness self-help workbooks will likely be increasingly more difficult (Van Zyl et al., 2019). But Internet-based 7-h interventions might be prohibitive without extensive use of the methods we identified above—frequent attention-grabbers, opportunities for feedback, and leveling-up rewards (Kelders et al., 2020). As an example, Nation et al. (2018) offered a free Internet-based REACH Forgiveness intervention lasting about 7 h. Only 26 percent of those who began the course completed it. Nation et al. (2018) speculated that people were used to coming to a Web site and working with it as long as they stayed engaged (i.e., 30 min, an hour, or two), but coming back to the Web site was difficult. That is, especially true without Web site designers building in automatic prompts to return delivered to responders’ email or text, and some system of monitoring and rewarding progress. Forgiveness, which has been found to be heavily dependent on time spent trying to forgive, might be most affected by the evolution of attention span and expectations in consumers of PPIs. More attention to these matters need to be considered in designing and implementing future forgiveness interventions.

#### Humility Interventions

Humility and patience have struggled to attract intervention researchers—for different reasons. Humility has struggled because people do not experience as much subjective discomfort from evaluating themselves as not humble as in evaluating themselves as unforgiving. Humility indeed has social benefits that have been well documented (see Worthington and Allison, 2018)—providing social oil that smooths relational conflict and promoting social bonds by being a social signal that one is other oriented. Yet, no clear connection has been made in the public mind that low humility is a problem, or at least is worthy of promotion (i.e., is related to other valued virtues or promotes wellbeing individually and socially). If humility research is to accelerate, this connection must become common belief that will not happen unless humility researchers make the empirical, theoretical, and publicly persuasive arguments to support it.

Furthermore, limited empirical and theoretical models exist that explain the routes toward humility. Despite being a valued virtue, there is little empirical evidence for the factors contributing to or influencing the development of humility (Watkins et al., 2016). Without a clear roadmap directing the route to the efficient practice of humility and without evidence-based interventions, interventionists are unable to generate valid and reliable intervention content that can promote humility confidently (Bollinger, 2018).

Despite this limitation, at least one of the two interventions aimed at developing humility showed promise. Both interventions employed traditional self-administered activity designs (such as workbooks), which were aimed at self-exploration. These interventions are self-paced and do not require the presence of a facilitator/therapist. However, these interventions relied on DIY workbooks. Although they were completed on a computer and submitted online, they we essentially traditional. Electronic formats (i.e., Apps, online interventions, and games) are needed to supplement the DIY workbooks and facilitated interventions must be developed for application in local communities if humility interventions are to be scaled.

Another element to consider is the paradoxical duality of humility. Humility is a personal function (or private experience) but can only exist (or be expressed) in the presence of others. Lavelock et al. (2014, p. 100) states that “the presence of others may present a paradox of toting one’s goodness for others to see and heightening one’s self-awareness, which is not ideal for seeking to transcend the self with humility”. However, humility can only be expressed in the presence of an active social context (Lavelock et al., 2017). Humility is therefore particularly difficult tos promote and develop (Lavelock et al., 2014). This duality should be directly addressed within humility interventions to enhance both the self as well as the self-in-relation to others. Furthermore, given the interpersonal nature inherent to humility, there is a clear need for developing interpersonal interventions that incorporate others within the person’s social system. Cuthbert et al. (2018) provided a start. They paired accountability partners to work through workbook interventions. More clever engagement of people’s social system are needed. Multi-modal intervention approaches could therefore be useful. These interventions would aim to develop holistic humility through a combination of self-administered intentional activities (e.g., self-help activities and workbooks), group developmental initiatives (e.g., training or positive growth groups), accountability partners, and individually focused interventions (e.g., strengths-based coaching) that can be facilitated both online and offline (Van Zyl and Rothmann, 2019; Van Zyl et al., 2020).

Finally, humility interventions should be expanded beyond the mere focus of general humility. Humility intervention researchers should develop intellectual humility (i.e., political humility and religious humility), spiritual humility (that is, humility in the face of God, nature, and humanity), relational humility (i.e., humility shaped differently for different types of relationships), and cultural humility interventions. Multi-facetted approaches toward humility development could therefore be greatly beneficial.

#### Patience Interventions

Patience interventions have been more frequent than humility interventions yet are plagued with similar issues. Additionally, patience interventions have almost exclusively been relegated to children, adolescents, and early adult college students (Schnitker et al., 2017b). For example, patience is often seen as something one grows into, and theoretical and empirical evidence exists that the impulsiveness of children and teens peaks at about age 17 or 18, yet the ability to control oneself cognitively rises almost linearly until it levels off about age 26. Thus, at some point between about 18 and 26, most young people will mature out of impatience—at least that is the theory (Schnitker and Westbrook, 2014; Schnitker et al., 2017b). As we all know, most adults frequently feel (and act) impatiently. Some adults are, by disposition, impatient. Yet, no interventions have been tested on adults except medical patients (Schnitker and Westbrook, 2014). Seeing patience as a virtue that will somehow right itself due to maturity has paralyzed research on middle-aged and older adults. Yet, that is clearly needed.

Moreover, despite the limited agreement on the components of patience, most interventions are exclusively focused on regulating behavior and emotional management. This has shown to be effective in certain population groups; however, the long-term sustainability thereof has been questioned (Schnitker and Westbrook, 2014). Patience interventions should supplement addressing the behavioral and emotional factors with patience interventions that focus on changing the attitudes or motivations for regulating behavior and emotions. Interventions aimed at changing motivations lead to more sustainable changes in behavior over time (Van Zyl and Rothmann, 2019).

Finally, interventionists and creators of interventions should be conscious of the “consumer culture” in mental health-related practices. People who need to build their patience would ironically be saying, “I want patience—now!” Creators of PPIs to promote patience must be cognizant of their audience’s inherent bias against sticking with patience interventions patiently—unless the creators heed new strategies to promote engagement (Schnitker et al., 2017b).

### How Do We Promote Growth in Temperance Intervention Research?

#### Funding for Research

One of the things that would increase the stable of researchers is additional grant funding. The turning point in research on forgiveness was the 1997 request for proposals that the John Templeton Foundation advanced. In the end, it funded about 20 research teams many of whom did multiple studies on forgiveness and that attracted post-doctoral researchers and graduate students who built their careers studying forgiveness. Money does not simply flow to a funding area. There must be a need and, following that, a funder whose priorities include building up the area. So what could create a need to study temperance?

#### Need for Interventions to Promote Temperance

One potential need could occur is there was a crisis associated with intemperance could increase research in temperance. As we found in the COVID-19 pandemic, crises mobilize action (Frenzel et al., 2021) and can result in amazing progress in intervention science. The development of numerous vaccines for COVID-19 over a single year was unprecedented. While no one wants some crisis to interfere with normal functioning, a public and widespread crisis of intemperance—such as an outcry against political polarization and a national or global outcry against injustice—could galvanize action in the research community.

As we saw in 2020, injustices are widespread throughout the world. Many of those might be thought to have occurred because people with abundant resources have pursued self-interested goals to the expense of less privileged people. Humility on behalf of the privileged might be needed to reverse this trend by not assuming that they have entitlement to an unfair share of resources. Forgiveness is needed by people who have been on the short end of social injustices instead of revenge that can perpetuate injustices. Forgiveness happens internally, as decisions to treat offenders as valuable people lead to emotional change that replaces negative unforgiving emotions with positive other-oriented ones (Worthington, 2020a). But, forgiveness does not negate one’s motivation to work for social justice, which can often be pursued more vigorously without having the demons of unforgiveness to keep under control. Worldwide justice movements might inspire the need for more research on temperance.

Some people believe that forgiveness by victims of social inequities might short-circuit the pursuit of social justice. However, forgiveness is not opposed to justice. In fact, justice is a social and societal phenomenon and forgiveness in internal, involving a decision to treat people less hatefully and more benevolently and an emotional transformation involving less rumination and thus less depression, anxiety, and anger. It is possible that victims’ forgiveness can remove people’s focus on themselves, or their expenditure of energy direct at rage and hatred, and thus leave them more able to pursue social justice. In addition, offenders and those who have the position to speak to historic wrongs from the offenders’ perspective need to be aware that their public statements of contrition, apology, and regret can encourage more forgiveness by victims, and amends making can begin or further reconciliation.

The awareness of increasing political and social polarization could create a sense of need to pursue a widespread building of temperance. This might involve political humility, forgiveness of people on the other side of the political spectrum, and patience as people wait for change to occur. Movements like global warming and environmental concern have been polarizing. If both sides could see the potential for temperance, it might lead to positive steps toward a healthier environment. In addition, geographic, financial evolution might create another sense of need for more global temperance. In Asia, China has risen as a technological, industrial, and financial power, and Japan has continued to be strong. In the Middle East, the worldwide weakening of oil dependence on Arab nations is likely to portend a shift in global economics. There will be ample opportunity to forgive any Arab nations that might have been perceived as holding economic power over much of the rest of the world. In Europe, the weakening of the European Union with the withdrawal of Great Britain provides opportunities to forgive both within the EU and Great Britain and in countries who have dealt with them. In South America, the economic and social strengthening of the southern hemisphere is a global trend. Some interventions have developed to treat unforgiveness that has come about from civil wars and other South American conflicts. Narvaez and Foundation ES.PE.RE is a noted example. In Africa, the rise in religious consciousness toward Christianity and in North Africa, Islam, have created the potential for religious tensions. These add to tribal tensions that have been long experienced in the Great Lakes region of East Africa, including Rwanda, Burundi, Uganda, and other countries. In Central America, emigration and political stabilization create needs for temperance—forgiveness, humility, and patience. In the United States, political polarization, conflict, the cancel culture, and divisions economically, politically, racially, and religiously are fertile grounds for practicing the virtue of temperance.

When people build temperance, they are not passively reacting to circumstances requiring restraint. Rather they are actively seeking to restrain negative knee-jerk reactions. Such restraint might take different forms. For example, people could tolerate changes. Tolerance involves restraining oneself from negative social responses, even though internally one might feel negative emotions. Forbearance involves restraining negative reactions for a positive motive—for the sake of group harmony. That positive motive can therefore mitigate the negative emotions of tolerance while yielding the same restraint of social wrongdoing as tolerance. Acceptance speaks to restraint of negative reaction and a passive internal emotional state. The position of acceptance might be actively arrived at yet there is more equanimity than with either forbearance or tolerance. Forgiving restrains negative reactions and also more actively puts the past in the past, yielding a different type of emotional equanimity than acceptance. Building forgiveness and having a patient stance toward seeing change come about socially, while having an attitude of humility, can provide people and communities resources to actively re-shape their interpersonal relationships in their communities to promote and sustain actions toward social change (Richter et al., 2021).

#### An Engaged Funder

Besides a need, research funding depends on an engaged funder. Government funding is likely if these social and economic, and racial pressures do not abate. Private funding from foundations and NGOs might also be aimed at these social tensions.

#### The Allure of New Technologies

New treatments inevitably provide, for some, an attraction of using a new technology to deliver the treatments. In addition, philosopher Peter Galison (1997) has shown that technological revolution can actually change the content of a science. Thus, as new technologies are developed, like apps that are immediately available, new interventions to promote patience, forgiveness, and humility might emerge. In 1957, Everett Rogers (2003) put forth a law of the diffusion of innovations: An innovation is disseminated by communication over time among a social system. When a new technology or treatment comes online, it is adopted in five waves. First, innovators begin using the technology. Then, early adopters jump in. Then, early majority adopters follow. Late majority adopters pick up the treatment. Finally, a laggard group joins in (just in time to miss the next new innovation).

- Knowledge leads. Often, innovators are drawn to initial knowledge, usually combined with internal drives to be seen as opinion leaders. Early knowledge is crucial to innovators’ adoptions. Yet most of the innovations fail. Thus, when innovators lead, usually at least some empirical success must attend their choice.
- Persuasion Cialdini (2016) suggests that persuasion to adopt something new (with at least a modicum of evidence) is based on six factors. One is reciprocity, feeling we owe the influencer something. Second, commitment and consistency speak to loyalty to existing products. Innovators must persuade early adopters to forswear their loyalty. Third, liking the influencer can influence early adopters (or any potential adopter) to take a chance. Fourth, endorsements by authorities, such as a recognized expert or (for younger generations) user-generated ratings of endorsement, can provide a sort of expertise. Often early adopters and early majority adopters usually rely on authority testimonies or word-of-mouth endorsements to adopt. Fifth, social proof involves seeing that numerous people are using a product successfully, which attracts late majority adopters and laggards. Sixth, scarcity can stampede adopters to make a positive decision at any point along the diffusion of innovations waves.
- Social confirmation. Once people have adopted a technology—whether innovator or laggard or anyone between—we all look for social confirmation that we have made the right choice. As cognitive psychologists tell us, we look to support our choices not disconfirm them (Kahneman, 2011).

## Conclusion

Because intervention research in temperance is in its infancy, the future looks rosy for PPI researchers. However, in this perspective paper, we suggest that we are in a second wave of PPI intervention research (Ivtzan et al., 2016) that requires a third-generation overhaul of the methods developed in the decades of the 2000s and 2010s. These new methods employed in the design of new interventions must draw on the empirical results of the previous two generations and yet be nimble and open to the new methods suggested by popular psychology as discerned from books, podcasts, and public talks. This includes being sensitive to the ironies of providing immediate gratification to people seeking to build their temperance.

In conclusion, to promote growth in temperance interventions, our lessons are few: First, new innovations must be developed—like creating temperance-related PPI apps, games, web-based brief interventions, engaging Internet-based interventions, and artificial intelligence interventions. Second, these innovations must be tested empirically for efficacy, effectiveness, dissemination, and scaling—each of which could necessitate modifications of content and delivery methods. Third, innovators must be attracted to the new methods, and the innovators must be likeable, have networks of loyal followers, and be persuasive enough to wean people from their existing commitments to temperance-focused evidence-based PPIs. Fourth, there must be initial positive data because the enthusiasm of innovators cannot carry a technology without some evidence of its efficacy. Fifth, leaders in the field must endorse the new intervention. Sixth, word-of-mouth communication of evidence that the new methods are successful in essential to build a following. Seventh, social proof and ultimately social confirmation are needed to sustain the use of the new innovations. Our fervent hope is that the present perspective article might inspire changes and provide ideas for new steps in building temperance by using PPIs.

## Data Availability Statement

The original contributions presented in the study are included in the article/supplementary material, further inquiries can be directed to the corresponding author.

## Author Contributions

EW conceptualized the paper. EW and LEvZ drafted and finalized the first version of the manuscript. All authors contributed to the article and approved the submitted version.

## Conflict of Interest

The authors declare that the research was conducted in the absence of any commercial or financial relationships that could be construed as a potential conflict of interest.

## Publisher’s Note

All claims expressed in this article are solely those of the authors and do not necessarily represent those of their affiliated organizations, or those of the publisher, the editors and the reviewers. Any product that may be evaluated in this article, or claim that may be made by its manufacturer, is not guaranteed or endorsed by the publisher.
